# Supplementary figures and images for: Bayesian Uncertainty-aware Deep Learning with noisy labels: Tackling annotation ambiguity in EEG seizure detection
Source: PLoS One. 2026 Jun 23;21(6):e0352191. doi: 10.1371/journal.pone.0352191 (PMC13289946; doi:10.1371/journal.pone.0352191)

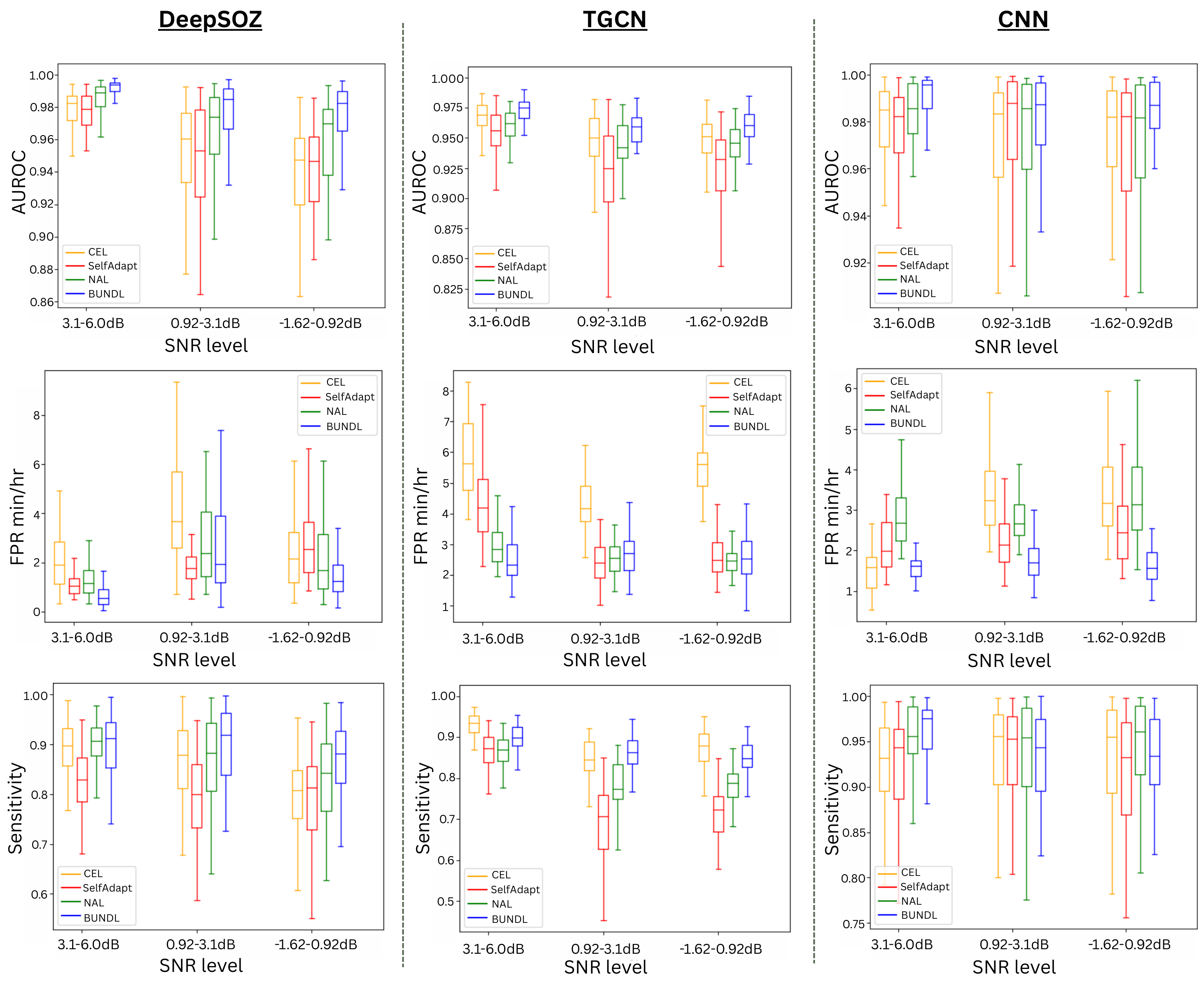

Supplement: S1 Fig — Box plots of AUROC, FPR (min/hour), and sensitivity metrics are shown from three deep networks at three different EEG signal-to-noise levels and symmetric noisy labels. Here the noise types indicates contamination in EEG itself and not labels. BUNDL maintains high AUROC compared to baselines despite higher noise interference in EEG. (TIFF) [file pone.0352191.s001.tiff]
